# Polymorphisms in microRNA target sites modulate risk of lymphoblastic and myeloid leukemias and affect microRNA binding

**DOI:** 10.1186/1756-8722-7-43

**Published:** 2014-06-02

**Authors:** Agnieszka Dzikiewicz-Krawczyk, Anna Macieja, Ewa Mały, Danuta Januszkiewicz-Lewandowska, Maria Mosor, Marta Fichna, Ewa Strauss, Jerzy Nowak

**Affiliations:** 1 Institute of Human Genetics, Polish Academy of Sciences, Strzeszyńska 32, 60-479 Poznań, Poland; 2 Faculty of Biology and Environmental Protection, University of Łódź, Pilarskiego 14/16, 90-231 Łódź, Poland; 3 Department of Medical Diagnostics, Dobra 38, 60-595 Poznań, Poland; 4 Department of Oncology, Hematology and Bone Marrow Transplantation, Poznań University of Medical Sciences, Szpitalna 27/33, 60-572 Poznań, Poland; 5 Department of Endocrinology and Metabolism, Poznań University of Medical Sciences, Przybyszewskiego 49, 60-355 Poznań, Poland

**Keywords:** microRNA-binding site polymorphisms, ALL, AML, CML, *ARHGAP26*, *ETV6*, *IRF8*, *PML*, *TLX1*

## Abstract

**Background:**

MicroRNA dysregulation is a common event in leukemia. Polymorphisms in microRNA-binding sites (miRSNPs) in target genes may alter the strength of microRNA interaction with target transcripts thereby affecting protein levels. In this study we aimed at identifying miRSNPs associated with leukemia risk and assessing impact of these miRSNPs on miRNA binding to target transcripts.

**Methods:**

We analyzed with specialized algorithms the 3′ untranslated regions of 137 leukemia-associated genes and identified 111 putative miRSNPs, of which 10 were chosen for further investigation. We genotyped patients with acute myeloid leukemia (AML, n = 87), chronic myeloid leukemia (CML, n = 140), childhood acute lymphoblastic leukemia (ALL, n = 101) and healthy controls (n = 471). Association between SNPs and leukemia risk was calculated by estimating odds ratios in the multivariate logistic regression analysis. For miRSNPs that were associated with leukemia risk we performed luciferase reporter assays to examine whether they influence miRNA binding.

**Results:**

Here we show that variant alleles of *TLX1*_rs2742038 and *ETV6*_rs1573613 were associated with increased risk of childhood ALL (OR (95% CI) = 3.97 (1.43-11.02) and 1.9 (1.16-3.11), respectively), while *PML*_rs9479 was associated with decreased ALL risk (OR = 0.55 (0.36-0.86). In adult myeloid leukemias we found significant associations between the variant allele of *PML*_rs9479 and decreased AML risk (OR = 0.61 (0.38-0.97), and between variant alleles of *IRF8*_ rs10514611 and *ARHGAP26*_rs187729 and increased CML risk (OR = 2.4 (1.12-5.15) and 1.63 (1.07-2.47), respectively). Moreover, we observed a significant trend for an increasing ALL and CML risk with the growing number of risk genotypes with OR = 13.91 (4.38-44.11) for carriers of ≥3 risk genotypes in ALL and OR = 4.9 (1.27-18.85) for carriers of 2 risk genotypes in CML. Luciferase reporter assays revealed that the C allele of *ARHGAP26*_rs187729 creates an illegitimate binding site for miR-18a-3p, while the A allele of *PML*_rs9479 enhances binding of miR-510-5p and the C allele of *ETV6*_rs1573613 weakens binding of miR-34c-5p and miR-449b-5p.

**Conclusions:**

Our study implicates that microRNA-binding site polymorphisms modulate leukemia risk by interfering with the miRNA-mediated regulation. Our findings underscore the significance of variability in 3′ untranslated regions in leukemia.

## Background

MicroRNAs (miRNAs) are a class of small (~22 nucleotide) nocoding RNAs that are potent regulators of gene expression in animals and plants. In animals miRNAs bind to target sequences (usually located in the 3′ untranslated region [3′UTR]) in messenger RNAs (mRNAs) and act by negatively regulating gene expression. This binding requires complementarity between the nucleotides 2-8 of miRNA (so called “seed” region) and the target mRNA [1]. To date more than 2500 mature human miRNAs have been identified [2] and they are predicted to regulate over 60% of human protein-coding genes [3]. This regulatory network can be very complex as one miRNA may potentially regulate several mRNAs, and a given mRNA may possess in its sequence binding sites for several miRNAs.

Since miRNAs control a wide variety of biological processes, including proliferation, apoptosis and differentiation, dysfunctions of the miRNA regulatory network may contribute to tumorigenesis. MiRNAs can act as both oncogenes and tumor suppressors. Examples of oncogenic miRNAs that are amplified or overexpressed in cancers include miR-17-92 cluster, miR-21, miR-155 and miR-372/373, while tumor suppressor miRNAs commonly deleted or with reduced expression in cancers are represented by miR-15a and miR-16-1, miR-34 family, let-7 family and miR-29 [4]. MiRNAs play a crucial role in normal hematopoiesis by controlling the differentiation of hematopoietic stem cells into different types of mature blood cells, while deregulation of miRNA networks has been linked to hematological malignancies [5]. Aberrant miRNA expression profiles have been observed in leukemias and lymphomas, and for several miRNAs there is experimental evidence for their functional involvement in leukemogenesis [6,7]. Specific miRNA expression signatures can accurately discriminate different leukemia subtypes and are often of great prognostic relevance [8-12]. Changes in miRNA expression may result from genomic and epigenetic alterations or the impairment of miRNA biogenesis pathway [13]. In addition, polymorphisms in miRNA genes or miRNA target sites (miRSNPs) can modify miRNA action. While polymorphisms in miRNA genes are relatively rare, SNPs in miRNA-binding sites in target genes are more frequent. Several studies have shown that SNPs in miRNA target sites enhance or weaken the interaction between miRNA and its target transcripts and are associated with cancers and other diseases [14,15]. In leukemia, however, so far only one study associated a SNP in the 3′UTR of the *NPM1* gene with adverse outcome in acute myeloid leukemia [16].

Considering that miRNAs have been shown to play an essential role in leukemogenesis and that SNPs in miRNA-binding sites in target genes have been associated with various cancers, in this study we aimed at identifying miRSNPs associated with leukemia risk and assessing the impact of these miRSNPs on miRNA binding to target transcripts.

## Results

### Characteristics of the study groups

Mean age at diagnosis was 7.2 years (range 0-17) for ALL patients, 51.6 (16-90) for AML patients, 51.5 (16-83) for CML patients and 51.4 (18-80) for controls % of males was 53% for ALL patients, 45% for AML patients, 54% for CML patients and 55% for controls. Clinical characteristics of leukemia patients are presented in Tables 1, 2 and 3.

**Table 1:** Clinical characteristics of ALL patients

**Parameters**	**ALL (n = 101)**
**Gender**	
Male	54 (53.5%)
Female	47 (46.5%)
**Mean age (years, range)**	7.2 (0-17)
**Initial WBC (×10**^**9**^**/L)**	
<20	63 (62.4%)
20- < 100	26 (25.7%)
≥100	12 (11.9%)
**Immunophenotype**	
pro-B-ALL	3 (3%)
pre-B-ALL	18 (17.8%)
common-ALL	63 (62.4%)
mature B-ALL	3 (3%)
T-ALL	14 (13.8%)
**FAB**	
L1	65 (64.4%)
L1/L2	5 (4.9%)
L2	28 (27.7%)
L3	3 (3%)

**Table 2:** Clinical characteristics of AML patients

**Parameters**	**AML (n = 87)**
**Gender**	
Male	39 (45%)
Female	48 (55%)
**Mean age (years, range)**	51.6 (16-90)
**FAB**	
M1	18 (20.7%)
M2	21 (24.1%)
M3	16 (18.4%)
M4	14 (16.1%)
M5	15 (17.2%)
Unknown	3 (3.4%)
**WHO classification**	
AML with t(8;21)	3 (3.4%)
APL with t(15;17)	16 (18.4%)
AML with inv16 or t(16;16)	5 (5.7%)
AML with t(9;11)	2 (2.3%)
AML with t(6;9)	1 (1.1%)
AML with myelodysplasia-related changes	27 (31%)
AML, not otherwise specified (NOS)	30 (34.5%)
Unknown	3 (3.4%)

**Table 3:** Clinical characteristics of CML patients

**Parameters**	**CML (n = 140)**
**Gender**	
Male	76 (54%)
Female	64 (46%)
**Mean age (years, range)**	51.5 (16-83)
**Cytogenetics**	
t(9;22) only	122 (87.1%)
trisomy 8	3 (2.1%)
del Y	5 (3.6%)
complex karyotype	5 (3.6%)
other abnormalities	4 (2.9%)
unknown	1 (0.7%)

### Identification of putative SNPs affecting miRNA binding

To identify putative miRSNPs we analyzed SNPs located in the 3′UTR regions of genes with reported relevance for leukemias (according to Entrez Gene). Out of 137 analyzed genes 3′UTRs of 88 genes did not harbor any SNP or SNP with minor allele frequency (MAF) greater than 0.05 in Caucasian population. The remaining 49 genes possessed 160 SNPs in their 3′UTRs (Table 4). These SNPs were analyzed using miRanda [17], PITA [18], Patrocles [19] and PolymiRTS [20] regarding their potential impact on miRNA binding. We identified 111 putative miRSNPs, of which 10 were chosen for further studies. The criteria for inclusion were: 1) concordance of at least two applied algorithms regarding the effect of the SNP on miRNA binding (except for rs2735383 G > C in *NBN* which was earlier associated with lung cancer and shown to affect binding of miR-629 [21]), and 2) expression of predicted miRNAs in bone marrow, white blood cells or leukemias and lymphomas according to the mimiRNA database [22] (Table 5).

**Table 4:** Leukemia-associated genes with polymorphisms in 3′UTR (minor allele frequency in caucasians > 0.05)

**Gene symbol**	**Gene name**	**No. of SNPs in 3′UTR**	**Gene symbol**	**Gene name**	**No. of SNPs in 3′UTR**
*ABL1*	c-abl oncogene 1, non-receptor tyrosine kinase	4	*KRAS*	Kirsten rat sarcoma viral oncogene homolog	9
*ABL2*	c-abl oncogene 2, non-receptor tyrosine kinase	4	*LMO2*	LIM domain only 2 (rhombotin-like 1)	1
*ACOXL*	acyl-CoA oxidase-like	2	*LPP*	LIM domain containing preferred translocation partner in lipoma	4
*ARHGAP26*	Rho GTPase activating protein 26	3	*MLF1*	myeloid leukemia factor 1	4
*ARNT*	aryl hydrocarbon receptor nuclear translocator	1	*MLLT11*	myeloid/lymphoid or mixed-lineage leukemia (trithorax homolog, Drosophila); translocated to, 11	1
*ATM*	ataxia telangiectasia mutated	4	*NBN*	nibrin	4
*BAX*	BCL2-associated X protein	1	*NEDD4*	neural precursor cell expressed, developmentally down-regulated 4, E3 ubiquitin protein ligase	2
*BCL2*	B-cell CLL/lymphoma 2	8	*NF1*	neurofibromin 1	3
*BCL2L11*	BCL2-like 11 (apoptosis facilitator)	2	*NQO1*	NAD(P)H dehydrogenase, quinone 1	1
*BCL3*	B-cell CLL/lymphoma 3	1	*P2RX7*	purinergic receptor P2X, ligand-gated ion channel, 7	1
*BCR*	breakpoint cluster region	8	*PBX1*	pre-B-cell leukemia homeobox 1	2
*BRCA2*	breast cancer 2, early onset	6	*PML*	promyelocytic leukemia	4
*C12orf5*	chromosome 12 open reading frame 5	11	*RUNX1*	runt-related transcription factor 1	1
*CCND1*	cyclin D1	4	*RYR2*	ryanodine receptor 2 (cardiac)	2
*CCND2*	cyclin D2	4	*SEPT9*	septin 9	3
*CEBPA*	CCAAT/enhancer binding protein (C/EBP), alpha	2	*SETBP1*	SET binding protein 1	1
*DEK*	DEK oncogene	1	*SKA1*	spindle and kinetochore associated complex subunit 1	
*ETV6*	ets variant 6	7	*STRN4*	striatin, calmodulin binding protein 4	1
*IKZF1*	IKAROS family zinc finger 1 (Ikaros)	5	*TAL1*	T-cell acute lymphocytic leukemia 1	4
*IRF1*	interferon regulatory factor 1	1	*TCF3*	transcription factor 3	2
*IRF4*	interferon regulatory factor 4	12	*TCL1B*	T-cell leukemia/lymphoma 1B	1
*IRF8*	interferon regulatory factor 8	5	*TLX1*	T-cell leukemia homeobox 1	5
*KDSR*	3-ketodihydrosphingosine reductase	2	*ZBTB16*	zinc finger and BTB domain containing 16	1
*KIT*	v-kit Hardy-Zuckerman 4 feline sarcoma viral oncogene homolog	2	*ZNF230*	zinc finger protein 230	1
*KMT2A*	lysine (K)-specific methyltransferase 2A	1			

**Table 5:** Candidate SNPs in miRNA target sequences

**Gene symbol**	**SNP ID**	**Predicted effect on miRNA binding**			
		**miRanda**	**PITA**	**Partocles**	**PolymiRTS**
*ABL1*	rs7457 C > T	***miR-23a/b ↑***	***miR-23a/b ↑***		***miR-23a/b ↑***
		**miR-96 ↑**			miR-323 ↑
*ARHGAP26*	rs187729 T > C	miR-579 ↑		***miR-18a/b ↑***	***miR-18 ↑***
*ATM*	rs227091 T > C	***miR-510 ↑***	**miR-214 ↑**	***miR-510 ↑***	***miR-510 ↑***
		miR-665↑	***miR-510 ↑***		**miR-512-5p ↑**
			miR-665↑		
			**miR-744 ↓**		
			**miR-939 ↓**		
*ETV6*	rs1573613 T > C	***miR-34a/c ↓***	***miR-34a/c ↓***	***miR-34b ↓***	***miR-34a/b/c ↓***
		***miR-449a/b ↓***	miR-885-3p ↓		***miR-449b ↓***
		miR-1207-5p ↓	miR-1207-5p ↓		
			**let-7b ↓**		
*IRF4*	rs1877176 G > A	**miR-101 ↑**		***miR-429 ↑***	**miR-141 ↓**
		**miR-107 ↓**			**miR-200b/c ↓**
		**miR-141 ↓**			***miR-429 ↑***
		miR-338-5p ↑			
		**miR-369-3p ↑**			
		***miR-429 ↑***			
*IRF8*	rs10514611 C > T	***miR-330-3p ↑***		***miR-330-3p ↑***	***miR-330-3p ↑***
		miR-562 ↑			
*NBN*	rs2735383 G > C	**miR-499-5p ↑**			
		miR-509-5p ↑			
		**miR-629 ↓**			
*PML*	rs9479 G > A	**miR-24 ↓**	**miR-383 ↑**	***miR-589-3p ↓***	***miR-589-3p ↓***
		***miR-510 ↓***	***miR-510 ↓***		
		miR-602 ↓	**miR-513a-5p ↓** miR-1182↑		
*TLX1*	rs1051723 C > T	***miR-539 ↑***		***miR-539 ↑***	***miR-539 ↑***
		miR-1300 ↓			
*TLX1*	rs2742038 C > T	miR-591 ↑	***miR-492 ↑***		***miR-492 ↑***

Predicted impact of the variant allele on miRNA binding by different algorithms: ↑ binding enhanced, ↓ binding weakened, miRNAs expressed in bone marrow, white blood cells or leukemias and lymphomas are in bold, miRNAs predicted by more than one algorithm are in italics.

### Effects of miRSNPs on leukemia risk

The selected miRSNPs were genotyped in leukemia patients and healthy individuals to identify miRSNPs associated with leukemia risk. Table 6 summarizes the genotype frequencies in the control and leukemia groups. All SNPs were in Hardy-Weinberg equilibrium in controls except for *ABL1*_rs7457 C > T, but for this SNP we did not detect significant associations with any leukemia type. The genotype frequencies of three SNPs were significantly different in the ALL patients compared with controls. *TLX1*_rs2742038 C > T and *ETV6*_rs1573613 T > C were associated with an increased risk of ALL in the recessive model (adjusted OR (95% CI) = 3.97 (1.43-11.02), p = 0.0081 (corrected p = 0.0356) and 1.9 (1.16-3.11), p = 0.0107 (corrected p = 0.0356), respectively), while *PML*_rs9479 G > A was associated with a decreased ALL risk in the additive model with adjusted OR (95% CI) = 0.55 (0.36-0.86), p = 0.0079 (corrected p = 0.0356). *PML*_rs9479 G > A was also associated with a decreased AML risk in the additive model (adjusted OR (95% CI) = 0.61 (0.38-0.97), p = 0.0372 (corrected p = 0.372). Two SNPs showed significant association with an increased CML risk in the recessive model: *ARHGAP26*_rs187729 T > C and *IRF8*_rs10514611 C > T with adjusted OR (95% CI) = 1.63 (1.08-2.47), p = 0.0213 (corrected p = 0.123) and 2.4 (1.12-5.15), p = 0.0246 (corrected p = 0.123), respectively. However, associations in AML and CML were significant only considering the uncorrected p-value. In ALL and CML more than one SNP was found to be associated with leukemia risk so we assessed effects of combined risk genotypes. In ALL risk genotypes were defined as *ETV6*_rs1573613 CC, *PML*_rs9479 GG, *TLX1*_rs2742038 TT, *ATM*_rs227091 CC and CT, and *IRF8*_rs10514611 TT (the latter two reached borderline significance in the analysis of individual SNPs). In CML risk genotypes were defined as *ARHGAP26*_rs187729 CC and *IRF8*_rs10514611 TT. In both leukemia types we observed a trend for an increasing leukemia risk as the number of risk genotypes rose, with OR for carriers of 3 or 4 risk genotypes in ALL reaching 13.91 (4.38-44.11) and for carriers of 2 risk genotypes in CML amounting to 4.9 (1.27-18.85) (Table 7).

**Table 6:** Distribution of genotypes of 10 selected miRSNPs in cases and controls and their association with leukemia risk

**SNP**	**Genotype**	**Controls (n = 471)**	**ALL (n = 101)**			**AML (n = 87)**			**CML (n = 140)**		
		**n (%)**	**n (%)**	**Adjusted**^**a **^**OR (95% CI)**	**p value**	**n (%)**	**Adjusted**^**b **^**OR (95% CI)**	**p value**	**n (%)**	**Adjusted**^**b **^**OR (95% CI)**	**p value**
				**Genetic model**	**(Corrected)**		**Genetic model**	**(Corrected)**		**Genetic model**	**(Corrected)**
***ABL1*** **_rs7457 C > T**	CC	*402 (85.3%)*	86 (85.1%)	1.3174 (0.2688-6.4561)	0.7339	78 (89.7%)	0.5735 (0.2744-1.1984)	0.1392	111 (79.3%)	1.5191 (0.9371-2.4627)	0.0898
	CT	*62 (13.2%)*	13 (12.9%)	recessive	(0.7858)	9 (10.3%)	additive	(0.5367)	27 (19.3%)	dominant	(0.2993)
	TT	*7 (1.5%)*	2 (2%)			0 (0%)			2 (1.4%)		
***ARHGAP26*** **_rs187729 T > C**	TT	110 (23.4%)	24 (23.75%)	1.0728 (0.6463-1.7807)	0.7858	26 (30.2%)	0.6946 (0.4173-	0.161	30 (21.4%)	**1.6297 (1.0755-2.4695)**	**0.0213**
	CT	255 (54.1%)	53 (52.5%)	recessive	(0.7858)	40 (46.5%)	1.1562)	(0.5367)	65 (46.4%)	recessive	(0.123)
	CC	106 (22.5%)	24 (23.75%)			20 (23.3%)	dominant		45 (32.2%)		
***ATM*** **_rs227091 T > C**	TT	151 (32.2%)	21 (22.6%)	1.6473 (0.9892-2.7430)	0.0551	27 (31%)	0.7452 (0.3866-1.4366)	0.3799	48 (34.3%)	0.9103 (0.6104-1.3573)	0.6446
	CT	236 (50.3%)	52 (55.9%)	additive	(0.1377)	48 (55.2%)	recessive	(0.5922)	67 (47.9%)	dominant	(0.8057)
	CC	82 (17.5%)	20 (12.5%)			12 (13.8%)			25 (17.8%)		
***ETV6*** **_rs1573613 T > C**	TT	158 (33.7%)	*35 (34.7%)*	**1.8994 (1.1606-3.1085)**	**0.0107**	26 (30.2%)	1.2629 (0.7114-2.2421)	0.4254	44 (31.4%)	0.7416 (0.4325-1.2715)	0.2771
	CT	229 (48.8%)	*37 (36.6%)*	recessive	**(0.0356)**	42 (48.9%)	recessive	(0.5922)	77 (55%)	recessive	(0.5542)
	CC	82 (17.5%)	*29 (28.7%)*			18 (20.9%)			19 (13.6%)		
***IRF4*** **_rs1877176 G > A**	GG	331 (70.3%)	75 (74.3%)	0.7758 (0.4792-1.2560)	0.3017	60 (69%)	1.4661 (0.4608-4.6649)	0.5170	101 (72.1%)	1.2897 (0.4478-3.7139)	0.6373
	AG	127 (27%)	25 (24.7%)	additive	(0.3771)	23 (26.4%)	recessive	(0.5922)	34 (24.3%)	recessive	(0.8057)
	AA	13 (2.7%)	1 (1%)			4 (4.6%)			5 (3.6%)		
***IRF8*** **_rs10514611 C > T**	CC	280 (59.6%)	56 (56%)	2.1876 (0.9232-5.1836)	0.0753	50 (57.5%)	1.1150 (0.7093-1.7526)	0.6373	84 (60%)	**2.4008 (1.1189-5.1514)**	**0.0246**
	CT	172 (36.6%)	36 (36%)	recessive	(0.1656)	33 (37.9%)	additive	(0.6373)	44 (31.4%)	recessive	(0.123)
	TT	18 (3.8%)	8 (8%)			4 (4.6%)			12 (8.6%)		
***NBN*** **_rs2735383 G > C**	GG	184 (39.2%)	32 (31.7%)	1.4140 (0.9089-2.2000)	0.1245	40 (46.5%)	0.7450 (0.4678-1.1863)	0.2148	56 (40%)	1.0854 (0.6492-1.8147)	0.7548
	CG	214 (45.5%)	50 (49.5%)	additive	(0.1778)	32 (37.2%)	dominant	(0.537)	61 (43.6%)	recessive	(0.8387)
	CC	72 (15.3%)	19 (18.8%)			14 (16.3%)			23 (16.4%)		
***PML*** **_rs9479 G > A**	GG	113 (24%)	37 (36.6%)	**0.5527 (0.3569-0.8560)**	**0.0079**	30 (34.5%)	**0.6055 (0.3778-0.9706)**	**0.0372**	37 (26.4%)	0.7626 (0.4759-1.2221)	0.26
	AG	246 (52.2%)	43 (42.6%)	additive	**(0.0356)**	39 (44.8%)	additive	(0.372)	76 (54.3%)	recessive	(0.5542)
	AA	112 (23.8%)	21 (20.8%)			18 (20.7%)			27 (19.3%)		
***TLX1*** **_rs1051723 C > T**	CC	224 (47.5%)	56 (56%)	0.7097 (0.4672-1.0782)	0.1081	38 (43.7%)	1.1586 (0.7294-1.8405)	0.533	66 (47.1%)	1.0198 (0.7076-1.4696)	0.9164
	CT	208 (44.2%)	37 (37%)	additive	(0.1778)	43 (49.4%)	dominant	(0.5922)	62 (44.3%)	additive	(0.9164)
	TT	39 (8.3%)	7 (7%)			6 (6.9%)			12 (8.6%)		
***TLX1*** **_rs2742038 C > T**	CC	325 (69%)	61 (60.4%)	**3.9718 (1.4314-11.0211)**	**0.0081**	58 (66.7%)	1.9108 (0.5019-7.2750)	0.3424	97 (69.3%)	0.3696 (0.0464-2.9453)	0.3472
	CT	137 (29.1%)	33 (32.7%)	recessive	**(0.0356)**	26 (29.9%)	recessive	(0.5922)	42 (30%)	recessive	(0.5787)
	TT	9 (1.9%)	7 (6.9%)			3 (3.4%)			1 (0.7%)		

For each SNP the best genetic model is presented.

OR, odds ratio; CI, 95% confidence interval. OR adjusted for ^a^sex and ^b^sex and age. Statistically significant results are in bold. Genotype frequencies in italics deviate from the Hardy–Weinberg equilibrium. Benjamiani-Hochberg false discovery rate control was used to correct for multiple comparisons.

**Table 7:** Cumulative effect of risk genotypes

**Leukemia**	**Number of risk genotypes carried**	**Controls (n = 468) n(%)**	**Patients n (%)**	**Adjusted OR (95% CI)**	**p value**
ALL^a^	0	91 (19.4%)	5 (5.4%)	ref.^c^	
n = 93	1	234 (50%)	47 (50.5%)	**3.67 (1.41-9.53)**	**0.0327**
	2	126 (26.9%)	28 (30.1%)	**4 (1.48-10.75)**	**0.0004**
	3 or 4	17 (3.6%)	13 (14%)	**13.91 (4.38-44.11)**	**<0.0001**
CML^b^	0	348 (74.4%)	88 (62.9%)	ref.^d^	
n = 140	1	116 (24.8%)	47 (33.6%)	**1.6 (1.06-2.41)**	**0.0261**
	2	4 (0.8%)	5 (3.6%)	**4.9 (1.27-18.85)**	**0.0209**

OR, odds ratio; CI, 95% confidence interval. Statistically significant results are in bold.

^a^Risk genotypes defined as *ETV6*_rs1573613 CC, *PML*_rs9479 GG, *TLX1*_rs2742038 TT, *ATM*_rs227091 CC and CT, and *IRF8*_rs10514611 TT.

^b^Risk alleles defined as *ARHGAP26*_rs187729 CC and *IRF8*_rs10514611 TT.

^c^OR adjusted for sex.

^d^OR adjusted for sex and age.

### Impact of SNPs on miRNA binding

For the miRSNPs that were associated with leukemia risk we performed luciferase assay to examine whether they influence binding of miRNAs predicted by the applied algorithms. No significant differences in luciferase levels were observed between the 3′UTR-wild type and 3′UTR-variant constructs for each 3′UTR tested in the presence of the miRNA negative control.

**Figure 1:**
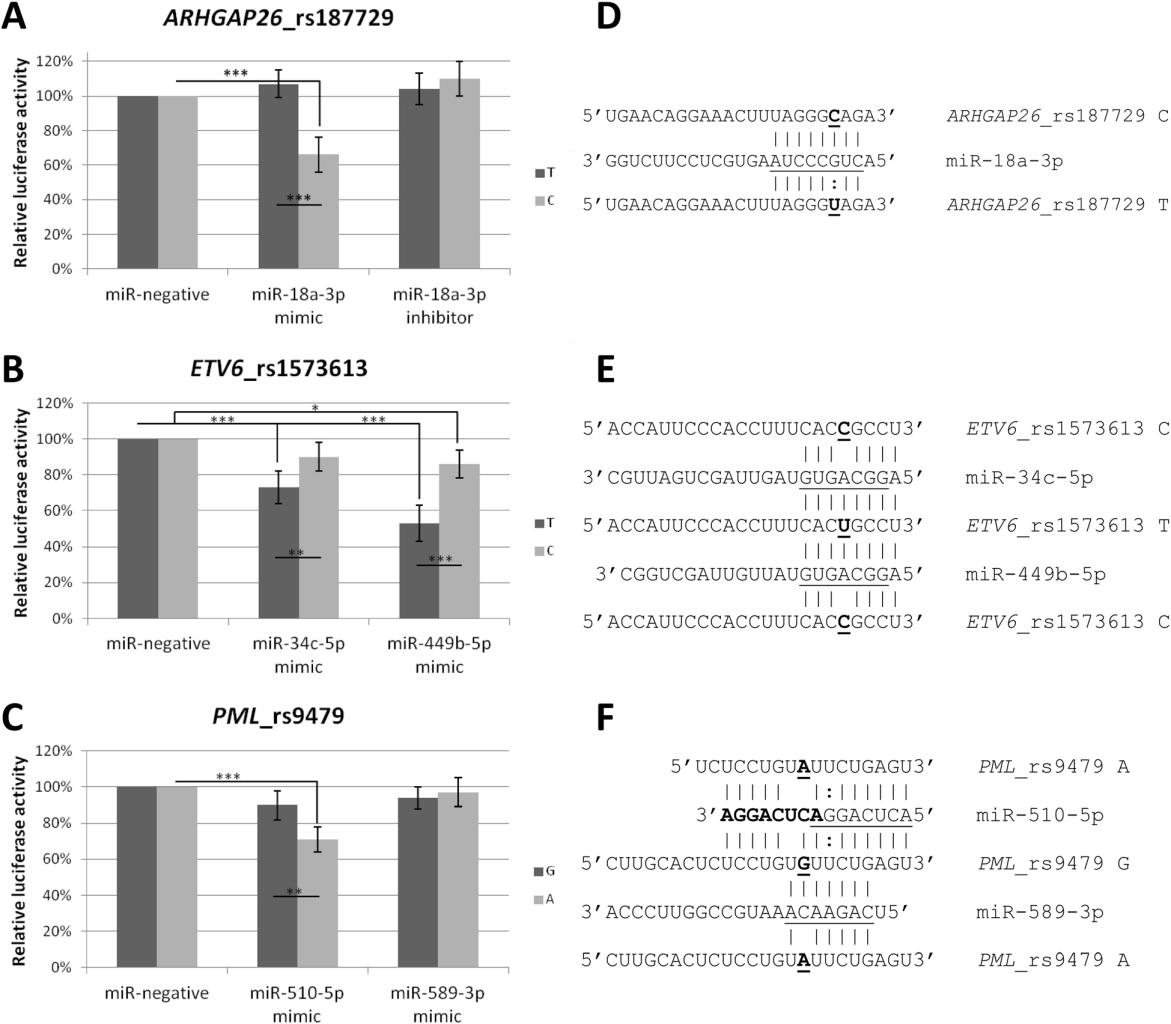
**Effect of miRSNPs on miRNA binding and protein expression. A-C)** Jurkat cells were transfected in triplicate with 1 µg either empty psiCheck2 vector or psiCheck2 constructs containing 3′UTRs with wild-type and variant alleles, with miRNA mimics, inhibitors or miRNA negative control (50 pmol/well). 24 h post transfection luciferase activity was measured. Data show relative *Renilla* luciferase levels normalized to firefly luciferase and corrected for the effect of miRNA mimics on the empty psiCheck2 vector. Values for the miRNA negative control were set as 100%. All transfections were done three times. * p < 0.05, ** p < 0.01, *** p < 0.001. Sequence alignments of **D)** miR-18a-3p with the *ARHGAP26* 3′UTR, **E)** miR-34c-5p and miR-449b-5p with the *ETV6* 3′UTR and **F)** miR-510-5p and miR-589-3p with the *PML* 3′UTR. MiRNA seed sequences are underlined. Allelic variants in each 3′UTR are underlined and in bold. For miR-510-5p its seed sequence repeated twice is shown (one in bold, the other underlined) instead of the entire mature miRNA sequence to show its complementarity with two adjacent miR-510 binding sites in the *PML* 3′UTR.

The C allele of *ARHGAP26*_rs187729 T > C replaces a G:U wobble with a canonical G-C pair (Figure 1B). This created a new binding site for miR-18a-3p (luciferase activity increased by 7% for the T allele and decreased by 34% for the C allele) (Figure 1A). MiR-18a-3p inhibitor only slightly increased luciferase level for the C allele (by 10%), suggesting that endogenous miR-18a-3p levels in Jurkat cells are too low to exert a significant regulatory effect on the *ARHGAP26* 3′UTR.

The C allele of *ETV6*_rs1573613 T > C introduces a mismatch in the centre of the target site for miR-34c and miR-449b-5p (these two miRNAs have the same seed sequence) (Figure 1D). This weakened binding of miR-34c-5p (luciferase activity reduced by 27% for the T allele and 10% for the C allele) and also decreased binding of miR-449b-5p (luciferase activity reduced by 47% for the T allele and 14% for the C allele) (Figure 1C).

The A allele of *PML*_rs9479 G > A introduces a mismatch within the binding site for miR-510-5p. The effect was, however, opposite to the prediction as the A allele enhanced binding of miR-510-5p (luciferase activity reduced by 10% for the G allele and 29% for the A allele) (Figure 1E). A detailed analysis of the *PML* 3′UTR sequence showed that only 7 bp downstream of the miR-510-5p binding site for the G allele of rs9479 (position 2621 of the 3′UTR) there is a second binding site for miR-510-5p (position 2628) (Figure 1F). Thus, in the 3′UTR with the G allele there are two sites competing for miR-510-5p binding: one in the position 2621 of the 3′UTR and second in the position 2628. Their close proximity may result in less efficient miR-510-5p binding to either site and decrease the regulatory effect on *PML*. Whereas in the 3′UTR with the A allele miR-510-5p may bind only to the position 2628, causing PML downregulation. The A allele of *PML*_rs9479 G > A also introduces a mismatch within the binding site for miR-589-3p (Figure 1F) but no significant effect on luciferase activity was observed for either variant of the *PML* 3′UTR (Figure 1E).

The T allele of *TLX1*_rs2742038 C > T was predicted to enhance binding of miR-492 and the T allele of *IRF8*_rs10514611 C > T was predicted to increase binding of miR-330-3p but we did not observe any noticeable effects on luciferase levels for either 3′UTR variant of those SNPs.

## Discussion

In this study we show that polymorphisms in microRNA-binding sites (miRSNPs) modulate leukemia risk and influence binding of miRNAs to target transcripts. To our knowledge this is only the second study reporting a relevance of miRSNPs in leukemia. Recently, Cheng et al. [16] identified a SNP in the 3′UTR of the *NPM1* gene that, although present with similar frequency in the control group, was associated with adverse outcome and shorter survival in patients with AML. They further showed that this SNP created an illegitimate binding site for miR-337-5p, which reduced levels of *NPM1* mRNA and protein. MiRSNPs in *PLA2G2A, IL-16* and *NOD2* were also studied in acute leukemia but no significant differences in the genotype frequencies between leukemia patients and control group were detected [23].

Our study identified five miRSNPs associated with risk of different leukemia types, which is in line with findings in other tumors showing that polymorphisms in miRNA-binding sites may predispose to cancer [21,24-26]. Moreover, we observed a significant trend for an increasing ALL and CML risk with the growing number of risk genotypes, indicative of a possible additive effect of the identified miRSNPs. This finding suggests that a panel of miRNA-binding site polymorphisms could be of clinical utility as markers of leukemia risk. To verify this possibility, miRSNPs reported in this study should be tested in prospective studies on independent patient groups.

In chronic myeloid leukemia SNPs in *ARHGAP26* and *IRF8* showed significant association with an increased risk of CML, however only for the uncorrected p-value. ARHGAP26 (GRAF) belongs to a family of Rho GTPase activating proteins and is a tumor suppressor acting by negatively regulating RhoA, a small GTP-binding protein with a growth-promoting effect in RAS-mediated malignant transformation [27,28]. Abnormal methylation of the *ARHGAP26* promoter and downregulation of the *ARHGAP26* mRNA was observed in acute myeloid leukemia and myelodysplastic syndrome [29,30]. In our study, the C allele of *ARHGAP26*_rs187729 T > C, associated with an increased risk of CML, created a new binding site for miR-18a-3p which decreased the protein expression by 34%. MiR-18a is a component of the oncogenic miR-17-92 cluster which is often amplified in aggressive B-cell lymphomas [31] and increased expression of miR-18a was correlated with a shorter overall survival in diffuse large B-cell lymphoma patients [32]. Our results demonstrate that the functional polymorphism in the 3′UTR of the tumor suppressor *ARHGAP26* creates an illegitimate binding site for miR-18a-3p, which may affect protein levels and contribute to an increased risk of CML.

A significant association with increased CML risk was also observed for a SNP in *IRF8*. IRF8 (ICSBP) is a transcription factor that regulates expression of genes stimulated by interferons and is essential for the differentiation of myeloid, dendritic and B-lymphoid lineages [33]. Its tumor suppressor activity is supported by mice with a null mutation of *IRF8* developing a CML-like syndrome [34] and lack of IRF8 expression in patients with chronic and acute myeloid leukemia [35]. The mechanism for the role of *IRF8* downregulation in the pathogenesis of CML is the regulation by IRF8 of several apoptosis-related genes [36,37]. We did not observe any effect of the *IRF8* 3′UTR on the protein expression in the presence of miR-330-3p, irrespective of the allele. However, it is possible that this SNP affects binding of another miRNA or influences other regulatory functions of the 3′UTR. The potential functional significance of *IRF8*_rs10514611 C > T remains to be elucidated.

In pediatric acute lymphoblastic leukemia we identified three miRSNPs modulating the ALL risk, in *ETV6, PML* and *TLX1*. The tumor suppressor ETV6 (TEL) is a transcription factor (repressor of translation) with a crucial role in the embryonic development and hematopoietic regulation [38,39]. Translocations involving the *ETV6* locus (12p13) are a frequent event in leukemia and myelodysplastic syndrome. They result in fusions with numerous partners and contribute to leukemogenesis by several pathogenic mechanisms [40]. In our study, the C allele of *ETV6*_rs1573613 T > C, associated with an increased risk of ALL, weakened binding of miR-34c-5p and miR-449b-5p, which resulted in higher protein levels (respectively 17% and 33% higher than for the T allele). The result is in accordance with the effect predicted by the *in silico* analysis, however it does not fit in the model of *ETV6* as tumor suppressor. A polymorphism associated with an increased leukemia risk would be expected to decrease rather than increase expression of a protein with a tumor suppressor function. Two cases of *ETV6* amplification have been described: one in B lymphoblastic leukemia [41] and the other in myelodysplastic syndrome [42]. Also in our study one patient with common-ALL had an additional chromosome 12 in her blast cell kariotype, and this girl was also a homozygote for the C allele of *ETV6*_rs1573613 T > C. It is possible then that *ETV6* can play a dual role of both a tumor suppressor gene and an oncogene, although a mechanism underlying the oncogenic activity of *ETV6* remains to be revealed. Among the known rearrangements involving *ETV6* there are a few in which the 3′UTR of *ETV6* is preserved. For example the MN1-ETV6 fusion protein acts as a transcriptional activator whereas ETV6 is a repressor, and the PAX5-ETV6 fusion protein is an aberrant transcription factor affecting both the PAX5 and the ETV6 pathways [40]. Increased expression of those fusion proteins resulting from the weaker binding of miR-34c-5p and miR-449b-5p to the 3′UTR with the C allele of *ETV6*_rs1573613 T > C could intensify their aberrant action. It is also feasible that the miRSNP in *ETV6* could act *in trans*. Recently a concept of “competing endogenous RNAs” (ceRNA) has been developed which assumes that messenger RNAs, transcribed pseudogenes and long non-coding RNAs compete for miRNA binding, intertwined in a large regulatory network [43]. Thus, presence of the C allele would release the miRNAs otherwise bound by the wild-type 3′UTR and increase the pool of miR-34c-5p and miR-449b-5p available for other targets. Indeed, members of the miR-34 family have been reported to be overexpressed in childhood ALL [44,45].

A significant association with elevated ALL risk was also observed for a SNP in *TLX1*. TLX1 (HOX11) is a transcription factor that plays a crucial role in embryonic development and in the genesis of the spleen [46]. Translocations involving the *TLX1* locus (10q24) and its increased expression occur in a significant proportion of T-ALL, supporting the oncogenic role of *TLX1*[47] and suggesting that *TLX1* expression in adult tissues is tightly controlled. We did not observe any effect of the *TLX1* 3′UTR on the protein expression in the presence of miR-492 for either allelic variant. Hence, the potential functional role of *TLX1*_rs2742038 C > T remains to be revealed.

The A allele of *PML*_rs9479 G > A was associated with reduced risk of both pediatric ALL and acute myeloid leukemia in adults. PML is a transcription factor and tumor suppressor that controls cell growth and apoptosis [48]. Translocation involving the *PML* locus (15q22) resulting in the expression of a fusion protein PML-RARα is found in the majority of acute promyelocytic leukemia cases [49]. Moreover, a partial or complete loss of the PML protein expression has been observed in several solid tumors [48], highlighting its role in cancerogenesis. In this study the A allele of *PML*_rs9479 G > A enhanced binding of miR-510-5p resulting in 19% decrease in the protein expression as compared to the G allele. However, lower expression caused by the A allele, which had a protective effect in our study, does not fit in the model of *PML* as a tumor suppressor. Increased expression of PML was observed in tumor cells of Hodgkin lymphoma [50] and hepatocellular carcinoma [51,52] suggesting an oncogenic role of PML but its functional significance is unknown. It is more plausible that the protective role of the A allele could be attributed to sequestering miR-510-5p from its other targets. Little is known about miR-510-5p function and its validated target genes but its oncogenic role was shown in breast cancer where overexpression of miR-510 increased tumor growth in vivo [53].

We are aware of some limitations of our study. The groups of leukemia patients were small, so the results should be treated as preliminary and need to be replicated in larger cohorts. Also, although we restricted our analysis to the miRNAs that are expressed in bone marrow, white blood cells or leukemias and lymphomas, the actual interaction of the analyzed miRNA-mRNA pairs in leukemic cells should be confirmed. Nonetheless, our study demonstrates that microRNA-binding site polymorphisms influence leukemia risk by interfering with the miRNA-mediated regulation of gene expression and underscores the significance of the variability in the 3′UTRs in leukemia.

## Methods

### Study groups

Since at the time when this study was conceived there were no reports on the association of miRNA-binding sites polymorphisms with leukemia, we decided to perform a pilot study in various leukemia types. The study comprised children diagnosed with acute lymphoblastic leukemia (ALL, n = 101) and adults with acute (AML, n = 87) and chronic myeloid leukemia (CML, n = 140) from oncology departments in Poznan, Poland. The control group (n = 471) was recruited among healthy blood donors with no history of cancer from Poznan Blood Centre. All protocols were carried out according to the Declaration of Helsinki. The study was approved by the Ethics Committee at the Poznan University of Medical Sciences (decision no 803/10) and informed consent was obtained from all subjects or their legal guardians.

### Selection of polymorphisms

We analyzed 137 genes associated with leukemia according to Entrez Gene [54] as of April 2011. As such we defined genes which in the field ‘Phenotypes’ in the gene report were linked with susceptibility to any type of leukemia. The 3′UTR sequences were obtained from the University of California Santa Cruz genome browser [55] in April 2011. SNPs residing in 3′UTR of these genes were identified by searching dbSNP [56]. SNPs with minor allele frequency (MAF) greater than 0.05 in Caucasian population were analyzed by specialized algorithms and databases: miRanda [17], PITA [18], the Patrocles database [19] and the PolymiRTS database [20] regarding their potential impact on miRNA binding.

### SNP genotyping

Genotyping was carried out on genomic DNA obtained from whole blood samples with TaqMan SNP Genotyping Assays (Applied Biosystems, Foster City, CA, USA) using the ABI PRISM® 7900HT Sequence Detection System and SDS 2.2 Software (Applied Biosystems). 15 ng of DNA was used per reaction. On each plate duplicate samples (5%), samples with known genotypes confirmed by direct sequencing and no template controls were run in parallel. The assays IDs are C__11160013_1_ (for *ARHGAP26*_rs187729), C___8866645_10 (for *TLX1*_rs1051723), C__30595554_20 (for *IRF8*_rs10514611), C___2280398_10 (for *ABL1*_rs7457), C___1576156_10 (for *IRF4*_rs1877176), C__16279225_10 (for *TLX1*_rs2742038), C__8899605_1_ (for *BCL11B*_rs1152781), C__2440679_10 (for *NBN*_rs2735383). *ETV6*_rs1573613 and *PML*_rs9479 were genotyped using Custom TaqMan SNP Genotyping Assays. The sequences for primers and probes are: for *PML*_rs9479 5′- TGCCCAAGAAAGAAACTTCTGTCA (forward primer), 5′- GCTATTGGCCAGGGACTCA (reverse primer), VIC-5′- CCTTGCACTCTCCTGTATT (probe 1), FAM-5′- CCTTGCACTCTCCTGTGTT (probe 2), and for *ETV6*_rs1573613 5′- GGCTGTGACAATTTACCGAAATGAT (forward primer), 5′- GCAAAAGGGAGTTCCTATTTCAAAAATGT (reverse primer), VIC-5′ CCACCTTTCACTGCCTAG (probe 1), FAM-5′- CACCTTTCACCGCCTAG (probe 2). *ATM*_rs227091 failed design of a custom TaqMan Assay and was therefore genotyped using the PCR-RFLP technique with the forward primer: 5′-GGCAAATTGTTCCAGGACAGC and reverse primer: 5′-AAGCCCTTCCCTTCCAACAG. PCR products were digested with *BsoBI* (New England Biolabs, Ipswich, MA, USA) and visualized on 2% agarose gel.

### Reporter constructs

SNPs for which we found statistically significant differences between the control and study groups were tested for their impact on miRNA binding by luciferase assay. 1000-1300 bp fragments of 3′UTRs were amplified from DNA of homozygotes for the major allele. The forward primer contained *XhoI* restriction site for convenient cloning. Purified PCR products were cloned into pGEM®-Teasy vector (Promega, Fitchburg, WI, USA). Inserts containing variant allele for each SNP were obtained by site-directed mutagenesis using the Quik-Change II Site-Directed Mutagenesis Kit (Stratagene, La Jolla, CA, USA). Wild-type and variant inserts were then cleaved out using *XhoI* and *NotI* (Promega) and subcloned into psiCheck2 vector (Promega) downstream the *Renilla* luciferase reporter gene. This vector contains also the secondary reporter, firefly luciferase, used for transfection normalization. Each construct was sequenced to confirm the sequence and orientation of the insert.

### Luciferase assay

Jurkat cells were plated on 24-well plate (2 × 10^5^ cells per well) in RPMI with 10% fetal bovine serum without antibiotics and transfected in triplicate with 1 µg either empty psiCheck2 vector or psiCheck2 constructs containing 3′UTRs with wild-type and variant alleles, with miRNA mimics or inhibitors or miRNA negative control (50 pmol/well) (Ambion, Austin, TX, USA) using Lipofectamine 2000 (Invitrogen, Carlsbad, CA, USA), according to the protocol. Among the studied miRNAs, only miR-18a-3p is expressed in Jurkat cells according to the mimiRNA database [22], and for this miRNA we also included its inhibitor in the luciferase assay. 24 h post transfection luciferase activity was measured using Dual-Luciferase® Reporter Assay System (Promega) according to the manufacturer’s protocol. All transfections were carried out three times.

### Statistical analysis

Hardy-Weinberg equilibrium of the genotypes in the study groups was verified by the Chi-square test. The association between SNPs and leukemia risk was calculated by estimating the odds ratio (OR) and its 95% confidence intervals (CI) in the multivariate logistic regression analysis, adjusted for sex and age. To detect the best genetic model (dominant, additive or recessive) first we performed the Cochran-Armitage test for trend and the model with the highest likelihood was chosen for the logistic regression analysis. Benjamini-Hochberg false discovery rate (FDR) control method was used to correct for multiple comparisons in each leukemia group. The SNPs showing a significant or borderline significant (before applying FDR) association with leukemia risk were then analyzed for the effect of the total number of risk genotypes on leukemia risk in multivariate logistic regression analysis, adjusted for sex and age. Only samples without any missing genotype were included in the analysis: n = 468 for controls, n = 93 for ALL and n = 140 for CML. Normalized *Renilla* luciferase reporter gene expression levels were compared by Student’s *t* test. P < 0.05 was considered statistically significant.

## Abbreviations

miRNA: microRNA; SNP: Single nucleotide polymorphism; miRSNP: miRNA-binding site polymorphism; ALL: Acute lymphoblastic leukemia; AML: Acute myeloid leukemia; CML: Chronic myeloid leukemia; 3′ UTR: 3′ untranslated region; MAF: Minor allele frequency; ceRNA: Competing endogenous RNA.

## Competing interests

The authors report no conflicts of interest.

## Authors’ contributions

ADK conceived the study, performed the experiments, analyzed the data, interpreted the results and wrote the manuscript. AM participated in the laboratory work. EM, MM and DJL recruited the patients. MF and ES recruited the control group. JN participated in the design of the study; DJL, MM and JN critically reviewed the manuscript. All authors read and approved the final manuscript.
